# Organizational commitment of health professionals and associated factors in primary healthcare facilities of Addis Ababa, Ethiopia: A multi-center cross-sectional study

**DOI:** 10.3389/fpubh.2022.981621

**Published:** 2022-10-17

**Authors:** Sulyeman Mohammed Arage, Derese Bekele Daba, Abere Yekoye Dessalegn

**Affiliations:** ^1^Department of Public Health, College of Medicine and Health Science, Werabe University, Warabe, Ethiopia; ^2^Department of Public Health, College of Medicine and Health Science, Ambo University, Ambo, Ethiopia; ^3^Departement of Midwifery, LeDeG Midwifery College, Addis Ababa, Ethiopia

**Keywords:** organizational commitment, work commitment, health professionals, primary health facilities, Addis Ababa

## Abstract

**Background:**

Organizational commitment has a positive impact on an organization's ability to provide professional services. Committed human power pushes an organization to achieve its goals, but non-commitment can lead to increased medical errors, prolonged inpatient admissions, and repeated hospitalizations leading to low quality of healthcare provision. However, to the best knowledge of researchers, there are no studies examining organizational commitment in the healthcare setting of Addis Ababa, Ethiopia.

**Objective:**

The aim of this study was to assess the level of organizational commitment and associated factors among health professionals working in the primary health facility of Addis Ababa, Ethiopia.

**Methods:**

A facility-based cross-sectional study was conducted among 459 healthcare professionals selected by simple random sampling from 12 health centers. Data were collected by three data collectors and one supervisor using a pretested questionnaire. Data were checked for completeness, cleaned, and entered into Epi-Data version 3.1.and exported into SPSS version 25 for analysis. In binary logistic regression statistical analysis, variables with *p* < 0.2 were entered in multivariate binary logistic regression analyses; then, the regression result was presented using COR, AOR with 95% CI, and a *p*-value < 0.05 as a level of significance.

**Result:**

The respondent's percent mean score of organizational commitment was 48.4%. Age group above 30 years (AOR = 1.52, 95% CI, 1.01, 2.30), those who were satisfied with their job (AOR 2.02, 95% CI 1.30, 3.13), and those who perceive good transformational leadership behavior (AOR: 1.85, 95% C.I, 1.18, 2.90) were significant factors of organizational commitment among health professionals.

**Conclusion and recommendation:**

Organizational commitment was lower in magnitude in the study setting. Age, job satisfaction, and transformational leadership behavior were significant predictors of organizational commitment.

## Background

In the 21st century, human resources and their performance are rendered as one of the most important factors for organizational success in achieving a set of organizational goals (1). Sustaining and improving the organization's ability to use human capital effectively and efficiently is a major challenge (2). Employee organizational commitment is one of the many obstacles that today's organizations face in terms of human resources (3). An accountable organization will also strive to have a positive work environment and ensure that the organizational framework and structure provide resources for employees' organizational commitment (4). The three dimensions that continue to limit the success of the development of universal health coverage by 2030 are availability, distribution, and performance of health workers (5). Ethiopia is one of the countries with a minimal health workforce with a density of 0.96 per 1,000 population. This is far below the African density of health workers (2.2/1,000 population and five times less than the minimum threshold of 4.45 per 1,000 population set by the World Health Organization (WHO) to meet the Sustainable Development Goal (SDG) health targets) (6). It is difficult to have an efficient and effective health system without a sufficient number of skilled, motivated, and supported health professionals. The involvement of highly skilled and committed health workforce is a critical component of the health system's performance (7).

The level of health professionals' organizational commitment was variable around the world. Studies from Urmia, Iran, Saudi Arabia, and the Philippines showed 67.3, 52.1, 3.13, and 63.9% of organizational commitment, respectively (8–11). Organizational commitment and job satisfaction in Zimbabwe, South Africa, and Namibia indicated that the mean score of organizational commitment was 4.76, 2.83, and 3.93, respectively (12). In Ethiopia, organizational commitment level varies from 32.9 to 74.6% (13–15).

Organizational commitment has both positive and negative consequences for organizations, especially in the case of the health sector. Health professionals with high commitment have characteristics of a strong desire to strive for achieving organizational goals, better job satisfaction, desire to stay in the organization, low absenteeism, low turnover intention, good mental spirit, increased effort and motivation, and retention in the organization by their sense of belongingness, affiliation, and attachment to organizations that leads to better organizational performance, their better manifestation of personal and organizational goals (16).

Contrary to the above points, employees with low organizational commitment have characteristics of low productivity and individual performance, feeling of discrimination in the workplace, ignorant of the needs of others, low participation in organizational matters, high absenteeism rate, low punctuality to the workplace, reduced interest to stay in the organization, high turnover, burnout, lack of trust, and motivation leading to work termination (17). Hence, all these preclude the organization from achieving its goals. In a healthcare setting, these lead to increased medical errors and increased and/or repeated hospitalizations. It also results in reduced patient satisfaction toward health services provided and elevated medical costs, cumulatively leading to interruptions of normal function. These ultimately result in a loss of organizational effectiveness and efficiency. In an organization where all these are common healthcare problems, it, finally, leads to low quality of healthcare (17–20).

In Ethiopia, to the extent of knowledge of the researcher so far, four published research works were found on the organizational commitment of health professionals. These were a study done in a Gurage zone (14), Bench Sheko zone (21), Jimma zone (15), and Jimma university teaching and specialized hospital (JUSTH) (13). Even though these studies showed important findings, they had certain limitations. The studies in Jimma and JUSTH focused on one profession: health professionals providing institutional delivery services and nurses, respectively. On the contrary, the study done in the Gurage zone had not assessed the effect of perceived leadership style on the organizational commitment of health professionals. A study done in the Bench Sheko zone included the effect of employee empowerment on the organizational commitment of health professionals, but all of them were not included the relationship between turnover intention and organizational commitment. It is also glanced that all the above four studies were conducted in the countryside and no sufficient studies are found in Addis Ababa, the capital city of Ethiopia.

Generally, even though a few studies are available in Ethiopia, majority are focusing on a single profession like the nurse profession or only localized to a single sphere, and still, there is a gap in showing organizational commitment and its predicting factors among healthcare professionals that are working in the primary healthcare setting, especially in the current study area. Furthermore, in the presence of a high prevalence of medical error (57.6%), high admission rate (74.7%), high turnover intention (77.5%), and so many problems that affect the quality of healthcare in Addis Ababa, still, no study shows the level of organizational commitment in this area (22–24). Therefore, the current study aimed to assess the level and factors affecting organizational commitment among health professionals working at primary health facilities in Addis Ababa, Ethiopia. The finding of this study will serve as an input in the development of successful change in management policies to improve job performance. It will also help as input for health policymakers to plan staff development activities, such as training, and decide remuneration scales to increase the level of commitment among health professionals.

## Methods

### Study design, setting, and period

An institution-based cross-sectional study was conducted among health facilities in Addis Ababa, Ethiopia. The proposed study was confined to Addis Ababa, the capital city of Ethiopia. There were eleven sub-cities of Addis Ababa city administration with a total population of 5,006,000. The study was conducted in primary public healthcare facilities of the Addis Ababa city administration. The city covers an estimated area of 527 Km^2^ with an estimated density of 5,165.1 people per square kilometer. The city has 11 sub-cities and 120 woredas. There are seven hospitals owned by the Addis Ababa health bureau, five hospitals owned by the Federal Ministry of Health, one hospital by Addis Ababa University, three hospitals by non-governmental organizations, three hospitals by the defense force, and 34 hospitals by private owners. There were 100 functional public health centers. There were a total of 7,486 health professionals working in primary public health facilities (25). This study tried to include professionals working in a health center. The study was conducted from 31 March to 15 April 2021.

### Population and eligibility criteria

The source populations were all health professionals working at primary public healthcare facilities (health centers) in Addis Ababa. Study populations were all sampled health professionals in a selected primary health facility that fulfilled inclusion criteria. The study unit was of randomly selected health professionals working at a randomly selected health center. Health professionals who were contract staff and those who were not available during data collection time due to annual leave, maternal leave, and sick leave were not involved in the study.

### Sample size determination, sampling technique, and sampling procedure

The sample size was determined using the single population proportion formula by considering the study done in Bench Sheko zone, SNNP region of Ethiopia using the following parameters; organizational commitment as 74.6% (21), 95% C.I, 5% margin of error, 5% non-response rate, and 1.5 design effect since the sampling technique was multistage. Based on all the assumptions, the final sample size was 459.

### Sampling procedure

A multistage sampling technique was used to select 459 study participants as follows. Out of eleven sub-city found in Addis Ababa city, 30% (4) sub-cities namely Gullele, Addis Ketema, Kolfe, and Kirkos sub-cities were selected by lottery method. Again, from each selected sub-cities, 30% of health centers were selected using a simple random sampling method. Proportional allocation of sample size was used for each randomly selected health center to select a sufficient amount of study subjects considering the total number of clinical staff in each health facility. Finally, the study subjects were selected by lottery method.

### Data collection tools and procedures

Data were collected using self-administered, structured, and pretested questionnaires. Validated tools were adapted from previously published pieces of literature (13–15, 21) which were checked for internal consistency of each question for a composite variable after the pretest. The questionnaire was divided into seven parts. Each part focus on socio-demographic variables, organizational commitment questions, job satisfaction, perceived organizational support questions, transformational leadership behaviors, perceived psychological empowerment, and turnover intention. Organizational commitment scale, perceived organizational support, transformational leadership behavior, and perceived psychological empowerment scale were addressed on a five-point Likert scale with response options ranging from 1 (very Disagree) to 5 (very Agree). A tool related to the job satisfaction scale was also addressed by a five-point Likert scale ranging from 1 (very dissatisfied) to 5 (very satisfied), while the turnover intention was addressed by agree (yes) and disagree (no) questions. The mean score for the level of organizational commitment was reported as the percentage of the score for a mean (%SM) after the calculation of the standardized mean value. It was calculated using the formula %SM = (Actual score –Potential minimum score/Potential maximum–Minimum)^*^ 100 (14).

### Variables and measurements

The dependent variable was an organizational commitment with a score ranging from 24 to 120. The mean overall organizational commitment scores were classified as low if it was below the means score and high if it was above the mean score. Job satisfaction score ranges from a minimum of 31 to a maximum of 155. It has sub-component scales like autonomy, professional opportunities, scheduling, pay and benefit, relation, and interaction. The higher the sum of the scores shows the higher (above the means core) and more satisfied with their job (15). Transformational leadership behavior measures individuals' opinions about how their immediate leaders were motivating and inspiring to them and how they were involved in setting good relationships. The score ranges from a minimum of 16–80. The higher the sum of the scores shows the higher (above the means core) the more good transformational leadership behavior. Perceived organizational support denotes the extent to which employees see that organizations recognize their contribution and care about their wellbeing. The score ranges from a minimum of 8–40. The higher the sum of the scores shows the higher (above the means core) had more good perceived organizational support. Perceived psychological empowerment denotes the extent to which organizations give authority and freedom to their employees to do formal tasks. The score ranges from a minimum of 6–30. The higher the sum of the scores shows the higher (above the means core) had more good perceived psychological empowerment. Turnover intention is an intention when a health professional in a healthcare facility is actively searching for a job during the data collection time or who is seriously thought about looking for a job in the last few months or who intends to leave the organization soon.

### Data quality assurance

The quality of data was maintained through different mechanisms. Tools were adapted from previously published and validated works. Two days of training were given to Diploma/ BSc nurses on the overall purpose and procedure of the study.

A pretest was done on 5% (*n* = 23) of the sample of health professionals working in Saris and Abinet Health centers (HC that were not considered for the actual data collection process). The supervisor was checking the completeness, consistency, and appropriateness of the collected data daily. The questionnaire was adjusted accordingly, and data were cleaned before entering into Epi-data software version 3.1 (The EpiData Association Odense, Denmark). Variable coding, cleaning, and recording were made through SPSS software (IBM Corporation, 2015).

### Data management and analysis

The data were coded and entered, and double-verified using EPI-DATA 3.1 software. Then, entered data were exported to SPSS version 25 for analysis. Descriptive statistics were used to summarize the data, and the results were presented using frequency tables, percentages, and graphs. Factors associated with the dependent variable were assessed using binary logistic regression. During bivariate analysis, variables with a *p*-value ≤ 0.25 were considered a candidate for multivariate logistic regression. Adjusted odds ratio (AOR) with 95% CI and *P*-value ≤ 0.05 were used to declare the presence of an association between explanatory variables and the level of organizational commitment of respondents. The Hosmer-Lemeshow goodness-of-fit test was applied to check model fitness.

## Operational definition

**Organizational commitment**: The score ranges from 24 to 120. The mean overall organizational commitment scores were classified as low if it is below the means score and high if above the mean score.

Job satisfaction means a positive or pleasurable emotional state resulting from the appraisal of one's job or job experience. It has subscales like autonomy, professional opportunities, scheduling, pay, and benefits. The score ranges from 31 to 155. The higher the sum of the scores shows the higher (above the means core) and more satisfied with their job (15, 21). Internal consistency of the variables was checked after pretest giving Cronbach's alpha (α) of 0.85.

Transformational leadership behavior measures individuals' opinions about how their immediate leaders were motivating and inspiring them and how they were involved in setting good relationships. The score ranges from 16 to 80. The higher the sum of the scores shows the higher (above the means core) better transformational leadership behavior having internal consistencies of 0.82.

Perceived organizational support indicates the extent to which employees see that organizations recognize their contribution and care about their wellbeing. The score ranges from 8 to 40. The higher the sum of the scores shows the higher (above the means core) had more good perceived organizational support having internal consistency of 0.87.

Perceived psychological empowerment indicates the extent to which organizations give authority and freedom to their employees to do formal tasks. The score ranges from 6 to 30. The higher the sum of the scores shows the higher (above the means core) had better perceived psychological empowerment (13, 21) having internal consistency of 0.79.

### Ethical consideration

Ethics approval was secured from Addis Ababa public health Research and Emergency management directorate with a reference number 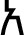/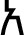/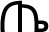/10507/227; then, a support letter was submitted to the selected sub-city Administration office and respective selected health center. The data were collected after the purpose or objective of the study was explained to each participant, and written informed consent was obtained by keeping COVID-19 mitigation protocols. Any personal identifying variables of each participant were kept confidential. To assure confidentiality, coding number was assigned to the study participants without mentioning their names.

## Result

### Socio-demographic characteristics of the participants

Among 459 questionnaires distributed, 453 were collected from the respondents (two questionnaires were unfilled, three questionnaires were not returned, and one questionnaire was incomplete) giving the response rate of the study to be 98.7%.

The participant's ages ranged from 21 to 58 years with a mean (±SD) score of 29.16 (±4.75) years, 60.9% were women, and more than half (57%) were married. Regarding their profession, 45.5% of them were nurses and 64.5% of participants were Bachelor's degree (BSc) holders. They had work experience ranging from 1 to 33 years with a mean (±SD) of 5.32 ± 3.55years. Their monthly salary ranges from 3,333 to 11,330 EBR with a mean of 6,711.34 (±1,692.36) EBR (Table 1).

**Table 1:** Socio-demographic characteristics of health professionals working in primary health facilities of Addis Ababa, Ethiopia, 2021.

**Variables**	**Categories**	**Frequency**	**Percentage**
Sex	Male	177	39.1
	Female	276	60.9
Marital status	Never married	195	43
	Married	258	57
Age	20–29	283	62.5
	≥30	170	37.5
Level of education	Diploma	141	31.1
	Bachelor degree	292	64.5
	Master's degree and above	20	4.4
Profession	Nurse	206	45.5
	Health officer	102	22.5
	Pharmacist	48	10.6
	Midwife	50	11
	Laboratory technician	28	6.2
	Others**	19	4.2
Work experience(years)	0–2	84	18.5
	3–5	182	40.2
	6–8	120	26.5
	≥9	67	14
Number of children	0	302	66.6
	≥1	151	33.4

Others^**^: medical doctors (GP), environmental health, and health extension.

### Level of organizational commitment

Organizational commitment percentage means the score of health professionals who participated in this study was 48.4%. On the contrary, the mean (±SD) raw score of this scale was 70.47 ± 13.77 ranging from 26 to 118. From the given organizational commitment items, 237 (52.3%) of the respondents scored a low level of organizational commitment and 216 (47.7%) of the health professionals scored a high level of organizational commitment.

### Level of job satisfaction

For the job satisfaction part, the total mean score was 53.7%. From the given job satisfaction items, 51.7% of the respondents were dissatisfied and 48.3% of the health professionals were satisfied with individual components of satisfaction percentage means to score and raw mean score are shown in Table 2 below.

**Table 2:** Job satisfaction items with respective mean and percentage scores of health professionals working in public primary health facilities of Addis Ababa, Ethiopia, 2021.

**Job satisfaction factors**	**Mean row score and SD**	**%SM**
Autonomy	13.28 ± 3.43	58.0
Professional opportunity	12.49 ± 3.89	53.1
Scheduling	16.97 ± 4.22	59.9
Support	22.04 ± 6.50	53.7
Pay and benefit	18.34 ± 7.10	40.5
Relationship and interaction	14.41 ± 3.76	65.0

### Level of transformational leadership behavior

The overall level of transformational leadership behavior (percentage means score) of health professionals who participated in this study was 53.1% (SM %) with a mean (±SD) raw score of 49.95 ± 13.15. From the given transformational items, 45% of the respondents had a poor perception of transformational leadership behavior while 55% of the health professionals had a good perception of transformational leadership.

### Level of perceived organizational support

The perceived organizational support percentage mean score was 52.6%, and the total mean (±SD) raw score was 24.82 ± 6.48. Almost half of the respondents (50.3%) had a good perception of organizational support, whereas 49.7% had a poor perception of organizational support.

### Level of psychological empowerment and turnover intention

Regarding perceived psychological empowerment, the total mean score was 65.4% with a total mean (±SD) raw score of 21.69 ± 5.20, while turnover intention total mean score was 66.5%. Of the total participant, 69.3% had an intention to leave their current organization.

### Predictors of organizational commitment

In a binary logistics regression model, age above 30, type of profession, number of children, job satisfaction, transformational leadership style, perceived organizational support, and turnover intention were assessed and became a candidate for multivariable logistic regression at *p*-value < 0.25.

### Independent predictors of organizational commitment

Those variables, which showed significant association with an organizational commitment of health professionals in bivariate binary logistic regression, were entered into the multivariable logistic regression and checked for multi-collinearity (by VIF and tolerance), normality (by histogram), and to what extent the model is good fitted (Hosmer-Lemeshow goodness of fit) and for those factors with *p* ≤ 0.05 considered as statistically significant as shown in Table 3 below.

**Table 3:** Predictors of organizational commitment with respective *p*-value, AOR, and confidence interval in multivariable regression of health professionals working in primary health facilities of Addis Ababa, Ethiopia, 2021.

**Variable**	**Categories**	**Organizational commitment**		**COR (95% C.I)**	**AOR**	**95% CI**	**P-V**
		**Not committed**	**Committed**				
Age	20–29 (Ref)	159	124				
	≥30	78	92	1.5 (1.03, 2.22)	1.52	(1.01,2.30)	0.047*
Profession	Nurse (Ref)	111	95				
	Health officers	47	55	1.37 (0.85–2.20)	1.35	(0.81, 2.25)	0.251
	Pharmacist	20	28	1.66 (0.87–3.09)	1.73	(0.88, 3.41)	0.113
	Midwife	34	16	0.55 (0.29–1.06)	0.62	(0.31, 1.23)	0.171
	Laboratory technician	14	14	1.17 (0.53–2.57)	1.08	(0.47, 2.48)	0.862
	Others	11	8	0.85 (0.33–2.2)	1.15	(0.43, 3.12)	0.780
Turn over intention	No(Ref)	85	54				
	YES	152	162	1.68 (1.12–2.52)	1.44	(0.93, 2.23)	0.098
number of children	0 (Ref)	165	137				
	≥1	72	79	1.32 (0.89–1.96)	1.06	(0.67, 1.66)	0.810
Job satisfaction	Unsatisfied (ref)	148	86				
	Satisfied	89	30	2.51 (1.72–3.67)	2.02	(1.3, 3.13)	0.002***
Transformational leadership	Poor (ref)	131	73				
	Good	106	143	2.42 (1.65–3.54)	1.85	(1.16,2.90)	0.008**
Perceive organizational support	Poor (ref)	133	92				
	Good	104	124	1.72 (1.19–2.50)	1.190	(0.768, 1.84)	
Perceive psychological empowerment	Poor (ref)	104	89				
	Good	133	127	1.12 (0.77–1.62)	0.77	(0.51, 1.18)	

Age (above 30) AOR (1.521, 95% C.I 1.008–2.295, *p*-value = 0.047), job satisfaction AOR 2.015 (95% C.I: 1.299–3.125, *P*-value = 0.002), and transformational leadership AOR (1.845, 1.175, 2.897) were statistically significant associated factors for good organizational commitment.

## Discussion

The major goal of healthcare organizations is to improve healthcare quality. To improve healthcare quality, it is highly detrimental to identify the level of organizational commitment, as well as contributing factors. As a result, this study was conducted to assess the level of organizational commitment and associated factors of health professionals working in a primary public healthcare facility in Addis Ababa, Ethiopia.

According to the current study, health professionals' organizational commitment was 48.4% (%SM). Overall, low commitment among healthcare professionals in Addis Ababa is a cause for concern, given that organizational commitment has implications for the efficiency, effectiveness, and sustainability of the health system.

The percentage mean score (%SM) of the current study was 48.4% which is in line with the study conducted in Saudi Arabia (47.88%) (8), but higher than a study done in Lahore (2.25) (26), Zimbabwe (2.83) (12), and Jima University Specialized Teaching Hospital where the raw means core was 70.45 ± 8.22 with only 72 (32.9%) of the nurses score high level of organizational commitment (13). In the case of Lahore (Turkey), the discrepancy might be due to their inclusion criteria difference (being large and research hospitals) and also they had used convenience sampling methods. In such large research hospitals, the workload might be high. Regarding the study done in Zimbabwe, the majority of employees were not permanent employees and were not unionized (27). As a result, they might deny telling real commitment level. The discrepancy observed from JUSTH was more likely explained by methodological differences employed in the sample size (*n* = 242), and the study participants were only nurses. In addition, the discrepancy might be attributed to the difference in study setting in that study that was conducted in a referral hospital tied to high workload which can greatly affect employee's commitment to the organization's interest.

Organizational commitment of the current study was lower than studies conducted in Iran (72.80 ± 4.95) (11), Portugal (3.97) (28), Gurage (64.81%) (14), Jimma (72.71%) (15), and Bench Sheko zone (74.6%) (21). The discrepancy might be justified by the fact that the study conducted in Iran used a small sample size, it focused only on emergency medical technicians, and the tool used to assess satisfaction was different from the one used in the current study. The study conducted in Portugal focused only on the affective component of organizational commitment, and it also included only nursing profession. In regards to the study conducted in a Gurage zone (14), the discrepancy could be due to the difference in the number of items and level of the Likert scale used to measure organizational commitment. In the current study, the organizational commitment was measured with 24 items on a five-point Likert scale, whereas a study conducted in the Gurage zone used nine items on a seven-point Likert scale. In terms of the study setting, the latter focused on both hospitals and health centers. However, since this study only emphasized health centers, the disparity may be attributed to the comparatively high incentive and different opportunities available in hospitals vs. health centers. The findings of the current study also differ from a study that was done at Jimma zone among health professionals that provide institutional delivery (15), and the discrepancy might be due to study participant difference which means only one profession was included. Regarding the latest study done in the Bench Sheko zone, organizational commitment in this study setting was higher than a finding of the current study, and the reason behind this might be the study area and socioeconomic status. When we see the study area of this finding, there were a lot of burdens other than a professional job. The payment might be not enough due to a lot of expenditures. The monthly income used for house rent, daily transportation, high cost of child education payment, etc. results in dissatisfaction and, in turn, results in a low commitment to their organization.

Among socio-demographic characteristics, the age of respondents had a significant association with organizational commitment. The age groups (30–39) were found to be significantly associated. This age commitment might be due to the age at family responsibility more rendered it but the low age group might be not such responsible. In contrast, older age groups who have worked for many years might be decreased their commitment due to burnout and they might be busy by having different responsibilities. This study finding was consistent with the study done in Iran with age group (31–40) but inconsistent with the finding in Gurage zone, JUSTH, and study done in Bench Sheko zone. The reason behind this might be due to the difference in the number of a participant in the age group. In this study, the number of participants in the age group of 30–39 was higher than in a study done in Gurage, JUSTH, and Bench Sheko zone (13, 14, 21).

This study also found a significant and positive association between job satisfaction and the organizational commitment of health professionals. The odds of having good organizational commitment were almost 2.02 times higher among those with job satisfaction than those without job satisfaction (AOR 2.02, 95% CI 1.30 and 3.13). This was the most important finding from this study which has support from theories. Accordingly, Frederick Herzberg's Two-Factor Motivation Theory is based on job satisfaction in the workplace. There are intrinsic factors that are related to job satisfaction, and extrinsic factors are associated with dissatisfaction. Meeting employees' lower-level needs (hygiene factors) by improving pay, benefits, and safety prevent employees from becoming dissatisfied but will not motivate them for better performance. To motivate workers, focus on changing the intrinsic nature and content of jobs themselves by enriching them to increase employees' autonomy and their opportunities to take on additional responsibility, gain recognition, and develop their skills and careers (29). Furthermore, Abraham Maslow's Hierarchy of Needs Theory strengthens this stating that satisfied employees will have a higher commitment to their organization (30).

This study also showed a significant association between organizational commitment and job satisfaction. The odds of organizational commitment were two times higher among those who were satisfied with their job than those who were less satisfied AOR 2.02 (95% C.I: 1.3–3.13). This was congruent with previous studies (Saudi Arabia, Iran, Philippines, Nigeria, Gurage zone, Jimma zone, and Bench Sheko zone) when job satisfaction of health professionals increases, their organizational commitment level also increases. From the job satisfaction component, pay and benefit and autonomy had a significant association with organizational commitment.

The other predictor that had a significant association with organizational commitment in this study was perceived transformational leadership behavior. The odds of having good organizational commitment were nearly two times higher among those with perceived transformational leadership style than those without perceived transformational leadership style (AOR: 1.85, 95% C.I: 1.18 and 2.90). This was in agreement with a study from Gurage (14), Jimma (15), and Bench Sheko zone (21) where the perceived transformational leadership style of health professionals was found to be a significant and strong predictor of organizational commitment. This could be due to the ability of transformational leaders to work with their followers or employees. Following this, a transformational leader is well-known for developing a futuristic plan, inspiring followers to achieve results beyond normally expected of them, and surpassing their interests for the goals of the organization (31). If health professionals are believed that they are not treated well by their immediate leader, they may believe they are not part of the organization and do not devote their time to the organization. Those health professionals who have positive perceived transformational leadership behavior of managers have an increased level of organizational commitment than those who have negative perceptions.

Perceived organizational support had a significant association in a study done in the Gurage zone, JUSTH, and Bench Sheko zone, but in this study, it had no significant association. This might be due to the difference in the number of managers and supervisors. In Addis Ababa, there are organized hierarchical management systems but in those areas mentioned above in most cases few managers and supervisors. The other factor might be socioeconomic and environmental conditions. In the case of the Gurage and Bench Sheko zone, the study setting had poor infrastructure such as transportation, and the supervisor might be not supported based on the standard, but in the case of JUSTH, the participant was a nurse and the profession by itself needs strict support from a supervisor.

## Limitation of the study

The finding of this study was limited to health centers. Therefore, the finding may not be generalized to health professionals working at hospitals. Since the study relied on the experience and exposure of health professionals, it might have recall bias and social desirability bias.

## Conclusion and recommendations

From this finding, it was concluded that the level of organizational commitment of health professionals working in primary health facilities of Addis Ababa was lower than what was reported in many other studies. Age of group of the participant, job satisfaction, and transformational leadership behavior were factors significantly associated with the organizational commitment of health professionals. Based on the study findings, the following recommendations to all concerned bodies.

## For policymakers

- Addis Ababa city administration, FMOH, Addis Ababa Health Bureau, each sub-city, and woreda health offices recommended maximizing the job satisfaction of their employees.
- For health, managers found Addis Ababa's primary health facility.
  - Health managers at all health department levels and health facilities are expected to adopt a transformational leadership style that is more attentive to health professionals' perceptions accordingly and also should have a special focus on the commitment of the under thirty age group.
- For researchers: since commitment level is a composite variable, further research is needed by including other antecedent factors which determine the organizational commitment of health professionals.

## Transparency statement

The leading author (DD) affirmed that the manuscript is an honest, accurate, and transparent account of the study being reported, that no important aspects of the study have been omitted, and that any discrepancies from the study as planned have been explained.

## Data availability statement

The original contributions presented in the study are included in the article/supplementary material, further inquiries can be directed to the corresponding author/s.

## Ethics statement

The studies involving human participants were reviewed and approved by Ethical approval was secured from Addis Ababa Public Health Research and Emergency management directorate with a reference number 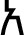/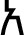/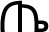/10507/227 then a support letter was submitted to the selected sub-city Administration office and respective selected health center. The patients/participants provided their written informed consent to participate in this study.

## Author contributions

SA and AD: conceptualization. SA and DD: data curation and formal analysis. SA, AD, and DD: methodology. DD and AD: supervision and validation. SA: original draft preparation. DD: writing, reviewing, and editing the manuscript. All authors contributed to the article and approved the submitted version.

## Conflict of interest

The authors declare that the research was conducted in the absence of any commercial or financial relationships that could be construed as a potential conflict of interest.

## Publisher's note

All claims expressed in this article are solely those of the authors and do not necessarily represent those of their affiliated organizations, or those of the publisher, the editors and the reviewers. Any product that may be evaluated in this article, or claim that may be made by its manufacturer, is not guaranteed or endorsed by the publisher.

## Abbreviations

AOR: adjusted odds ratio
CDC: communicable disease control
EPI INFO: epidemiology information
HAI: healthcare-associated infection
IP: infection prevention
PPE: personal protective equipment
SPSS: software package of social science
SSI: surgical site infection
USA: United States of America
WHO: World Health Organization.

## References

[1] Al-Jabari B Ghazzawi I. Organizational commitment: a review of the conceptual and empirical literature and a research agenda. Int Leadersh J. (2019) 11:78–119.

[2] Erdogan V Yildirim A. Healthcare professionals' exposure to mobbing behaviors and relation of mobbing with job satisfaction and organizational commitment. Proc Comput Sci. (2017) 120:931–8. 10.1016/j.procs.2017.11.328

[3] WHO. Monitoring the Building Blocks of Health Systems: A Handbook of Indicators and their Measurement Strategies. World Health Organization (2010).

[4] Tella A Ayeni C Popoola S. Work motivation, job satisfaction, and organizational commitment of library personnel in academic and research libraries in Oyo State, Nigeria. Library Philos Pract. (2007) 9:13. Available online at: https://digitalcommons.unl.edu/libphilprac/118

[5] Rahimifard S Trollman H. UN Sustainable Development Goals: An Engineering Perspective. Taylor & Francis (2018). p. 1–3.

[6] Haileamlak A. How can Ethiopia mitigate the health workforce gap to meet universal health coverage? Ethiop J Health Sci. (2018) 28:249. 10.4314/ejhs.v28i3.1 29983523 PMC6016355

[7] Mbindyo PM Blaauw D Gilson L English M. Developing a tool to measure health worker motivation in district hospitals in Kenya. Hum Resour Health. (2009) 7:1–11. 10.1186/1478-4491-7-40 19457237 PMC2692978

[8] Al-Haroon HI Al-Qahtani MF. Assessment of organizational commitment among nurses in a major public hospital in Saudi Arabia. J Multidiscip Healthc. (2020) 13:519. 10.2147/JMDH.S256856 32606723 PMC7320227

[9] Fabiene EE Kachchhap SL. Determinants of employee commitment among healthcare professionals. Int J Acad Res Account Finan Manag Sci. (2016) 6:44–52. 10.6007/IJARAFMS/v6-i2/2038

[10] Moradi Y Baghaei R Rahmani A Mollazadeh F. The association between organizational commitment and quality of services offered to nurses by a hospital: a descriptive-correlational study. J Occup Health Epidemiol. (2020) 9:35–40. 10.29252/johe.9.1.35

[11] Saberinia A Zadeh MA. Job satisfaction and organizational commitment: a study on emergency medical technician in the Southeast of Iran. Ann Med Health Sci Res. (2019) 723–28.

[12] Maleka M Mpofu M Hlatywayo CK Meyer I Carr S Parker J. Employee engagement, organizational commitment, and job satisfaction in Namibia, South Africa, and Zimbabwe: an exploratory study. J Psychol Africa. (2019) 29:393–400. 10.1080/14330237.2019.1647964

[13] Israel B Kifle W Tigist D Fantahun W. Organizational commitment and its predictors among nurses working in Jimma University specialized teaching hospital, Southwest Ethiopia. Primary Health Care Open Access. (2017) 7:1–8. 10.4172/2167-1079.1000262

[14] Nima GH Kerie MW Nebeb GT. Organizational commitment of health professionals and associated factors in government health facilities of the Gurage zone, South Ethiopia. Clin Med Res. (2016) 5:82–90. 10.11648/j.cmr.20160505.11

[15] Siraneh Y Ololo S Tsega G Yitbarek K Adamu A Erchafo B. Level and factors associated with professional commitment of health professionals providing institutional delivery services in public health facilities, Southwest Ethiopia. Ethiop J Health Sci. (2018) 28:495. 10.4314/ejhs.v28i4.15 30607062 PMC6308734

[16] ElDin YKZ Abd El Rahman RM. The relationship between nurses' perceived pay equity and organizational commitment. Life Sci J. (2013) 10:889–96.

[17] Chen S-Y Wu W-C Chang C-S Lin C-T Kung J-Y Weng H-C. Organizational justice, trust, and identification and their effects on organizational commitment in hospital nursing staff. BMC Health Serv Res. (2015) 15:1–17. 10.1186/s12913-015-1016-8 26347451 PMC4562203

[18] Bagraim JJ. Commitment and the emigration intentions of South African professional nurses. Health SA Gesondheid. (2013) 18:512–7. 10.4102/hsag.v18i1.512

[19] Luthans F Avolio B Avey J Norman S Norman S Child I. Proceedings of the International social sciences and tourism research conference 20-22 APRIL 2016 editors. J Bus Res. (2007) 30:121–33. Available online at: https://www.researchgate.net/publication/303518663

[20] Masud H Daud WNW. Human resource management practices and organizational commitment: research methods, issues, and future directions. Rev Integr Bus Econ Res. (2019) 8:217–26. 10.6007/IJARBSS/v8-i11/5159

[21] Alemayehu D Ololo S Siraneh Y. Organizational Commitment and Associated Factors Among Health Professionals Working in Public Health Facilities of Benchsheko Zone Southwest Ethiopia. Clin. Med. Res. (2021).

[22] Aniley AW Taye B Girma B. Magnitude of turnover intention and associated factors among nurses working in emergency departments of governmental Hospitals in Addis Ababa, Ethiopia: a cross-sectional institutional-based study. BMC Nursing. (2020) 19:97. 10.21203/rs.2.21566/v4 33071646 PMC7556577

[23] Bohn J Kassaye B Record D Chou B Kraft I Purdy J. Demographic and mortality analysis of hospitalized children at a referral hospital in Addis Ababa, Ethiopia. BMC Pediatr. (2016) 16:1–5. 10.1186/s12887-016-0709-4 27765020 PMC5073447

[24] Endalamaw A Dessie G Biresaw H Belachew A Amare D Workineh Y. Medication errors in Ethiopia: systematic review and meta-analysis. BMC Public health. (2020) 22:477. 10.21203/rs.3.rs-35808/v132296652

[25] Tsutsumi J Bendewald MJ. Urban environmental challenges in developing cities: the case of the Ethiopian capital Addis Ababa. Int J Environ Ecol Eng. (2010) 4:164–9. Available online at: http://scholar.waset.org/1307-6892/12541

[26] Gider Ö Akdere M Top M. Organizational trust, employee commitment and job satisfaction in Turkish hospitals: implications for public policy and health. East Mediterr Health J. (2019) 25:622–9. 10.26719/emhj.19.010 31625587

[27] Agency NS. The Namibia Labour Force Survey 2014 Report. Namibia: Namibia Statistics Agency (2017).

[28] Orgambídez A Almeida H. Predictors of organizational commitment in nursing: results from Portugal. Investig Educ Enfermerí*a*. (2018) 36:870–9. 10.17533/udea.iee.v36n1e14 29898353

[29] Yusoff WFW Kian TS Idris MTM. Herzberg's two factors theory on work motivation: does its work for today's environment. Global J Commer Manag. (2013) 2:18–22. Available online at: https://www.longdom.org/articles/herzbergs-two-factors-theory-on-work-motivation-does-it-work-for-todays-environment.pdf

[30] McLeod S. Maslow's hierarchy of needs. Business. (2018) 3–5. Available online at: https://canadacollege.edu/dreamers/docs/Maslows-Hierarchy-of-Needs.pdf

[31] Lai F-Y Tang H-C Lu S-C Lee Y-C Lin C-C. Transformational leadership and job performance: the mediating role of work engagement. Sage Open. (2020) 10:2158244019899085. 10.1177/2158244019899085

